# Season of Birth, Sex and Sleep Timing Preferences

**DOI:** 10.3390/ijerph120505603

**Published:** 2015-05-22

**Authors:** Yuee Huang, Dongdong Lin, Chuanwen Lu, Gholam Ali, James Metzger, Nivedita Shankar, Tan Xu, Wenjie Sun, Guangliang Shan

**Affiliations:** 1 Department of Preventive Medicine, School of Public Health, Wannan Medical College, Wuhu 241001, China; E-Mail: huangyewindow@163.com; 2 Laboratory for Environment and Health, School of Earth and Environment, Anhui University of Science and Technology, Huainan 231001, China; 3 School of Science and Engineering, Tulane University, New Orleans, LA 70112, USA; E-mail: dlin5@tulane.edu; 4 Department of Environmental Toxicology, The Institute of Environmental and Human Health, Texas Tech University, Texas Tech University Health Sciences Center, Lubbock, TX 79416, USA; E-Mail: lu.chuanwen@gmail.com; 5 School of Medicine, Tulane University, 1430 Tulane Ave, New Orleans, LA 70112, USA; E-Mail: gali1@tulane.edu; 6 Histecon Associates, Inc. Little Rock, AR 72205, USA; E-Mail: jemetzger@aristotle.net; 7 Saw Swee Hock School of Public Health, National University of Singapore, 117549, Singapore; E-Mail: nivedita_shankar@nuhs.edu.sg; 8 Department of Epidemiology, School of Public Health, Medical College of Soochow University, Suzhou 215123, China; 9 Department of Epidemiology, School of Public Health and Tropical Medicine, Tulane University, New Orleans, LA 70112, USA; 10 School of Food Science, Guangdong Pharmaceutical University, Zhongshan 528458, China; 11 School of Public Health and Tropical Medicine, Tulane University, New Orleans, LA 70112, USA; 12 Department of Epidemiology, Institute of Basic Medical Sciences, Chinese Academy of Medical Sciences, Beijing 100005, China; 13 School of Basic Medicine, Peking Union Medical College, Beijing 100005, China

**Keywords:** season of birth, sex, sleep timing

## Abstract

*Objective*: To evaluate whether the season of birth and sex are associated with preferences for bedtime among Chinese adults. *Methods*: A national population-based study on sleep preferences was conducted among Chinese in 2008. A questionnaire was used to collect information on the sleep time of Chinese adults. Analysis of covariance was used to examine the relationship between season of birth and preferences for bedtime. Two sets of potential confounders were used in the adjusted models. Model 1 adjusted for age. Model 2 additionally adjusted for area, occupation, education level, smoking, and drinking. *Participants and Measurements*: The questionnaire was administered to a sample of 3959 Chinese adults. *Results*: Men had a higher delayed mean sleep onset and offset time (22:38 and 6:32) than women (22:18 and 6:25). Men also slept for a shorter duration compared to women (7 h 54 min *vs.* 8 h 7 min). Women born in fall had the latest sleep onset time sleep offset time (22:23/6:30), compared to their counterparts born in winter. These associations were attenuated by additional adjustments of more confounders. *Conclusions*: There were significant differences in sleep timing preferences between men and women. Season of birth was not associated with sleep timing in Chinese adults.

## 1. Introduction

The duration and timing of sleep have been associated with both physical and mental health [1,2,3]. Sleep timing is an important aspect of sleep [4]. Individuals who consider themselves to be “morning persons” naturally prefer to go to bed early in the evening and wake up early in the morning whereas those who consider themselves to be “night owls” naturally prefer to go to bed late in the evening and wake up late in the morning. However, chronotype is not necessarily fixed and is subject to external factors as well. A study from Norway showed that sleep timing and chronotype are affected by changes in daylight hours; seasons with decreased daylight hours are associated with delayed sleep timing and chronotype compared to seasons with longer daylight hours [5].

The hypothesis that photoperiod at birth could act as a sort of imprinting phenomenon was put forward for the first time in 1999 [6]. Recently, Natale *et al*. tested the hypothesis by comparing people born in opposite hemispheres and observed that more “night owls” were born during the seasons associated with longer photoperiods (spring and summer), and more “morning persons” were born during the seasons associated with shorter photoperiods (autumn and winter), indirectly supporting an imprinting-like phenomenon played by the photoperiod at birth [7].

Season of birth, associated with health status and diseases [8,9], has been shown to be associated with sleep timing [10,11]. The possible explanation is based on the circadian typology theory that individuals born during the spring or summer have internal clocks with longer days than those born in the autumn or winter. This may potentially result in a phase delay for individuals born in the spring and summer and a phase advance in individuals born in the autumn and winter [12,13].

However, most studies on sleep timing were conducted in Western countries. Of note, Doi *et al*. studied the sleeping/waking patterns and circadian typology of Japanese preschoolers and found no correlation between season of birth and chronotype [14]. It was postulated that the difference in results between Doi *et al.* and previous studies was due to geographic, cultural, and ethnic differences in sleep patterns between Western and Eastern populations, a difference that has been documented in other studies [15]. The measurement of sleep pattern is different from the measurement of sleep-timing preference, although the two are highly correlated. Hareda *et al.* did find an association between season of birth and chronotype in Japanese children similar to that seen in other populations, though it was only present in the younger (2–12) and not older age (18–25) group [16].

To our knowledge, there has been no population-based study for sleep timing conducted in China until now. In the present study, we examined the relationship between sex, season of birth, and sleep timing in a community-based Chinese population. We presented two main hypotheses: (1) season of birth was associated with preferred sleep timing preferences; and (2) sleep timing preferences were varied by the season of birth.

## 2. Methods

### 2.1. Participants

The China Sub-Health Survey (CSHS) was initiated in 2008 and lasted for one month, with the aim to understand the health status based on a national sample. Details about the study design were reported in Sun *et al.* [17]. In brief, the CSHS selected individuals from six provinces and was designed to represent 1.4 billion Chinese. A multi-stage, random cluster sampling design was used to draw study subjects.

All of 31 provinces or municipalities were divided into six administrative regions (Northeast, North, East, Central South, Southwest, and Northwest), according to administrative geography. The areas of Jilin, Beijing, Jiangsu, Hubei, Chongqing and Gansu were selected randomly to represent those six administrative parts. The latitudes of each of the groups studied are: Jilin (40ʹ52ʺ–46ʹ18ʺ), Hubei (29ʹ05ʺ–33ʹ20ʺ), Chongqing (28ʹ10ʺ–32ʹ13ʺ), Jiangsu (30ʹ45ʺ–35ʹ20ʺ), and Beijing (39ʹ54ʺ), and Gansu (32ʹ31ʺ–42ʹ57ʺ). Then, each selected area was classified into urban and rural. We selected one or two urban and another one or two rural areas randomly. In those selected study areas, college students, local residents, local government staff, enterprise workers, and farmers were clustered and selected randomly as samples. In total, 19,665 participants were surveyed, and 18,630 responded and filled out the questionnaires effectively (response rate was 94.7%). We excluded individuals who: (1) were less than 20 or greater than 60 years of age; (2) had symptoms mental illness (based on the questionnaires) that could potentially affect sleep patterns; (3) were shift workers, and (4) did not have birth place information. Finally, 3959 participants were included in the analysis.

This study was approved by the Institutional Review Board at Peking Union Medical College and followed the tenets of the Declaration of Helsinki. Written informed consent was obtained from all participants.

### 2.2. Data Collection

Trained investigators explained details to participants on how to collect and record the data with a standard questionnaire by using the self-administrative method. Pertinent data that was collected included demographics, medical history, medications, preferences for bed time, wake-up time, and other information about sleep behavior.

### 2.3. Sleep-Timing Preferences

A questionnaire on sleep was translated and modified from the Horne-Ostberg [18] and Pittsburgh Sleep Quality Index [19]. Sleep-timing preferences were evaluated based on a subject’s preferred sleep onset time, preferred sleep offset time, preferred sleep duration, and midpoint of sleep. These time preferences were obtained from participants through items in the questionnaire that asked:

(1) Considering only your body’s own natural internal rhythm, if you had an entirely free day, at what time would you naturally wake up?
(2) Considering only your body’s own natural internal rhythm, if you had an entirely free evening, at what time would you naturally go to bed?

Preferred sleep duration and preferred midpoint of sleep time were calculated according to the following formulas:

Preferred sleep duration = (preferred sleep offset time + 24) − (preferred sleep onset time);

Preferred midpoint of sleep = [(preferred sleep offset time + 24) − (preferred sleep onset time)]/2 + (preferred sleep onset time)

In the analysis, preferences of sleep time was evaluated based on preferred onset time, preferred offset time, preferred duration and preferred midpoint.

### 2.4. Statistical Analysis

Sleep time preferences were analyzed as continuous outcome variables and season of birth was analyzed as a categorical variable. According to birth date, season of birth was categorized into four groups: spring (22 March to 22 June); summer (22 June to 22 September); fall (22 September to 22 December); and winter (22 December to 22 March). Winter was the reference group.

Analysis of covariance (ANCOVA) was used to examine the association between season of birth and preferences of sleep time by sex. Tukey’s *post hoc* test was further applied when ANCOVA was significant. Two sets of potential confounders were used in the adjusted models. Model 1 adjusted for age. Model 2 additionally adjusted for living conditions, area, occupation, education level, marital status, smoking, and drinking. All potential confounders are summarized in Table 1. All tests were two-sided and significance level was set at 0.05. SAS for Windows Statistical Software Package Version 10.0 (SAS Institute, Cary, NC, USA) was used for data processing and analysis.

**Table 1:** Demography and socioeconomic characters among Chinese adults by sex.

Characters	Men (n = 2161)	Women (n = 1798)
Age (year, means ± SD)	37.2 ± 10.1	34.7 ± 9.3
Birth Season (%)		
Spring	22.26	20.41
Summer	25.22	24.36
Autumn	27.67	28.53
Winter	24.85	26.70
Area (%)		
Jilin	15.78	16.91
Gansu	6.39	6.34
Chongqing	36.7	39.88
Jiangsu	14.35	14.74
Hubei	15.69	4.12
Beijing	11.1	18.02
Occupation (%)		
Civil	22.95	18.85
Profession	14.11	16.41
Worker	39.33	35.87
Famer	5.37	5.78
Business/service	9.21	11.18
Students	6.02	8.68
Others	3.01	3.23
Education (%)		
Liberate/primary school	2.93	5.41
High school	46.82	43.98
College	50.25	50.61
Drink (%)		
No	39.06	91.51
Yes	60.94	8.49
Smoke (%)		
No	46.01	98.33
Yes	53.99	1.67
Sleep onset time (means ± SD)	22:38 ± 1h08	22:18 ± 1h03
Sleep offset time (means ± SD)	06:32 ± 1h03	06:25 ± 0h58
Sleep duration (means ± SD)	7h54 ± 0h56	8h07 ± 0h52
Midpoint of sleep(means ± SD)	2:31 ± 0h51	2:16 ± 0h39

## 3. Results

There were 2161 men and 1798 women in the study. The average age was 37 years for men, and 35 years for women, respectively. The percentage of men born over the four seasons were 22.26%, 25.22%, 27.67%, and 24.85%, while 20.14%, 24.36%, 28.53%, and 26.70% were women. Table 1 shows the baseline demographics of our study population by sex. Most people were highly educated, non-smoker, and non-drinker. Men had a delayed mean sleep onset and offset time (22:38 and 6:32) than women (22:18 and 6:25).

**Table 2:** Means of the dependent variables taken into account, according to season of birth.

Characteristic	Spring		Summer		Fall		Winter	
	Men	Women	Men	Women	Men	Women	Men	Women
Sleep Onset Time	22:34 ± 1h08	22:16 ± 1h05	22:39 ± 1h07	22:18 ± 1h02	22:43 ± 1h07	22:23 ± 1h05	22:36 ± 1h11	22:14 ± 1h02
Sleep Offset Time	06:30 ± 0h54	06:20 ± 0h57	06:31 ± 1h03	06:26 ± 0h55	06:34 ± 1h06	06:30 ± 1h01	06:33 ± 1h07	06:23 ± 0h59
Sleep Duration	7:56 ± 1h07	8:03 ± 1h07	7:52 ± 1h07	8:08 ± 1h01	7:50 ± 1h12	8:07 ± 1h07	7:57 ± 1h11	8:08 ± 1h06
Sleep Midpoint	2:32 ± 0h52	2:18 ± 0h51	2:35 ± 0h56	2:22 ± 0h50	2:38 ± 0h56	2:27 ± 0h54	2:35 ± 0h59	2:18 ± 0h51

Notes: Adjust for age (as continuous variable), area, education, occupation, and smoking, drinking.

**Table 3:** Sleep timing preference for men by season of birth.

Characteristics	Spring		Summer		Fall		Winter		*p* for Trend
	β (95% CI)	*p*	β (95% CI)	*p*	β (95% CI)	*p*	β (95% CI)	*p*	
Sleep onset time									
Model1 ^a^	−0.05 (−0.19, 0.09)	0.49	0.05 (−0.09, 0.18)	0.51	0.09 (−0.04, 0.22)	0.17	0 (0, 0)	ref.	0.16
Model2 ^b^	−0.02 (−0.14, 0.11)	0.81	0.03 (−0.091, 0.15)	0.61	0.092 (−0.03, 0.21)	0.13	0 (0, 0)	ref.	0.08
Sleep offset time									
Model1 ^a^	−0.06 (−0.19, 0.06)	0.29	−0.03 (−0.15, 0.08)	0.57	−0.02 (−0.14, 0.1)	0.79	0 (0, 0)	ref.	0.71
Model2 ^b^	−0.03 (−0.14, 0.08)	0.62	−0.04 (−0.15, 0.07)	0.47	−0.03 (−0.13, 0.08)	0.63	0 (0, 0)	ref.	0.54
Sleep duration									
Model1 ^a^	−0.02 (−0.15, 0.12)	0.82	−0.08 (−0.21, 0.06)	0.25	−0.11 (−0.24, 0.03)	0.11	0 (0, 0)	ref.	0.40
Model2 ^b^	−0.01 (−0.15, 0.13)	0.86	−0.07 (−0.2, 0.06)	0.29	−0.12 (−0.25, 0.01)	0.07	0 (0, 0)	ref.	0.45
Midpoint of sleep									
Model1 ^a^	−0.06 (−0.16, 0.05)	0.31	0.01 (−0.1, 0.11)	0.92	0.04 (−0.06, 0.14)	0.47	0 (0, 0)	ref.	0.29
Model2 ^b^	−0.02 (−0.12, 0.07)	0.66	−0.003 (−0.10, 0.09)	0.94	0.03 (−0.06, 0.13)	0.47	0 (0, 0)	ref.	0.11

Notes: ^a.^ model 1 adjusts for age (as continuous variable); ^b.^ model 2 adjusts for age (as continuous variable), area, education, occupation, smoking, and drinking.

Men also slept for a shorter duration compared to women (7 h 54 min *vs.* 8 h 7 min, respectively). Compared with men, women preferred a significantly earlier sleep onset time, earlier sleep offset, and earlier midpoint of sleep (*p* < 0.05, data was not shown in Table 1).

Table 2 shows that in both sexes, people born in fall had the latest sleep onset time (22:43 for men and 22:23 for women), offset time (6:34 for men and 6:33 for women), and midpoint time (2:38 for men and 2:27 for women) while people born in spring had the earliest offset time (6:30 for men and 6:20 for women), and midpoint of sleep time (2:32 for men and 2:18 for women). Of note, men born in spring had the earliest onset time but not women (22:34 for men and 22:16 for women).

Table 3 shows that in model 1 after adjusting for age, there was no significant difference on sleep timing among different season of birth people. In model 2, further adjustment of the age (as continuous variable), area, education, occupation, smoking, and drinking did not change the results.

Table 4 shows that in model 1 after adjusting for age, women born in fall had a significantly later sleep onset time, sleep offset time, and midpoint of sleep time than those in winter (*p* < 0.05). In model 2, additional adjustments for potential confounders attenuate the association on the sleep onset and sleep offset, but do not attenuate the association on midpoint of sleep time.

**Table 4:** Sleep timing preference for women by season of birth

Characteristics	Spring		Summer		Fall		Winter		*p* for Trend
	β (95% CI)	*p*	β (95% CI)	*p*	β (95% CI)	*p*	β (95% CI)	*p*	
Sleep onset time									
Model1 ^a^	0.03 (−0.11, 0.17)	0.69	0.07 (−0.06, 0.21)	0.3	0.15 (0.02, 0.28)	0.03 *	0 (0, 0)	ref.	0.16
Model2 ^b^	0.08 (−0.04, 0.21)	0.19	0.07 (−0.04, 0.19)	0.23	0.12 (0.00, 0.23)	0.05	0 (0, 0)	ref.	0.09
Sleep offset time									
Model1 ^a^	−0.06 (−0.18, 0.64)	0.34	0.07 (−0.05, 0.19)	0.23	0.14 (0.02, 0.25)	0.02 *	0 (0, 0)	ref.	0.03
Model2 ^b^	−0.03 (−0.15, 0.08)	0.59	0.07 (−0.04, 0.18)	0.21	0.09 (−0.02, 0.19)	0.1	0 (0, 0)	ref.	0.008 ^†^
Sleep duration									
Model1 ^a^	−0.09 (−0.24, 0.06)	0.234	0.001 (−0.14, 0.14)	0.98	−0.01 (0.14, 0.12)	0.87	0 (0, 0)	ref.	0.69
Model2 ^b^	−0.12 (−0.26, 0.03)	0.11	−0.003 (−0.14, 0.13)	0.96	−0.03 (−0.16, 0.1)	0.66	0 (0, 0)	ref.	0.54
Midpoint of sleep									
Model1 ^a^	−0.02 (−0.13, 0.09)	0.77	0.07 (−0.03, 0.18)	0.18	0.14 (0.04, 0.25)	0.006 ^†^	0 (0, 0)	ref.	0.03 *
Model2 ^b^	0.03 (−0.07, 0.12)	0.6	0.07 (−0.02, 0.16)	0.13	0.1 (0.012, 0.19)	0.03 *	0 (0, 0)	ref.	0.006 †

*Notes*: ^a.^ model 1 adjusts for age (as continuous variable); ^b.^ model 2 adjusts for age (as continuous variable), area, education, occupation, smoking, and drinking. * *p* < 0.05;^†^ *p* < 0.01.

## 4. Discussion

Our results indicated that men had a later mean sleep onset, offset and midpoint time than women. There is no significant difference between the birth season and sleep onset, offset, or sleep duration in men and women, although there was a significant association between season of birth in women’s midpoint of sleep time.

Our results were not in line with Dillon *et al.*’s conclusion that women tended to have more intra- individual variability in sleep-onset latency than men [20]. The differences in results between Eastern and Western studies on sleep could be due to biological differences between Caucasians and Asians, like polymorphism of circadian clock genes [21] and ocular photosensitivity [22]. The potential national and cultural differences between populations could significantly affect sleep preferences [6].

Our results showed that there are no significant associations between season of birth and parameters of sleep time (sleep onset time, offset time, midpoint of sleep). Our results confirmed a non-significant relationship between season of birth and sleep duration, which is in line with previous studies [10]. Roenneberg *et al.* pointed out that sleep time and sleep duration were independent [23].

Regarding sleep timing, our results confirmed that season of birth was not associated with offset time, which was reported by Tonetti *et al.* in an adolescent population [11]. The possible explanation is that societal pressure is more likely to modify the sleep-offset time. Natale *et al.* analyzed the effect of season of birth on preferred sleep-wake cycle timing as assessed by Morningness-Eveningness Questionnaire among 5720 university students with mean age of 22 years. They pointed out that the students’ sleep offset time was more likely to be affected by the societal pressure (e.g., timing for school) than ideal personal rhythms and their results also showed the effect of season of birth on preferred sleep onset time [10].

The variation on sleep timing preference by season of birth could be explained by potential confounders e.g., health status. For example, Sun *et al.* conducted a hospital-based study on individuals in China with sleep disorders and found there was an association between insomnia and the month of birth [24]. However, our population was community-based and mostly healthy.

However, Harada *et al.* conducted a study on the effect of season of birth on people residing at low latitude area, Kochi, Japan (33° N). Their results show that the effect of season of birth on the “morning person” chronotype is only limited to young children aged 1–12 years, declining in older persons (13–25 years) [16]. Our participants are aged 20 years or above and residing at latitude of 28–46° N. Thus significant results on the mid-sleep on women in our study could be due to chance. It is more likely to get such “statistically” significant results in a large sample size study that compares two groups.

## 5. Limitations

There are several limitations to our study. First, this study was based on self-reported questionnaires. Therefore, more objective quantitative measures will be preferred and included in our further research design. Second, we cannot rule out the possibility of residual confounding, such as BMI [25,26] and obstructive sleep apnea, since we did not have such information. Third, the start times of work/study of survey participants were not available in this study. Future studies with that information are warranted.

## 6. Conclusions

Our results suggest that there is a significant difference in sleep timing preference by sex but not season of birth.
